# The Hospital World

**Published:** 1910-03-05

**Authors:** 


### The Hospital World.

### A Fund for Injured Doctors.

At the last meeting of the Executive Board of the Manchester Royal Infirmary an interesting announcement was made. The Finance Committee reported that under the will of the late Mr. G. W. Smith a sum of £2,339 8s. 1d. had been bequeathed to the hospital to form the nucleus of a fund to be devoted, at the discretion of the Medical Board, to the assistance of any member of the medical or surgical staffs of the hospital suffering illness or otherwise incapacitated through injuries sustained in the course of hospital work. The testator appears to have had more particularly in mind injuries resulting from accidents at post-mortem examinations or operations, but full discretion has been left to the Medical Board to use the money as may be thought fit. This is, we believe, the only institution that possesses such a fund specially set aside for such cases.

### Personalia.

At the annual meeting of the Malton Cottage Hospital Earl Fitzwilliam was re-elected President.

Sir George White, Bart., has been re-elected President of the Bristol Royal Infirmary.

Lord Hothfield, Mr. C. E. Homewood, Mr. G. Ashley Dodd, the Rev. A. H. Harrison, Mr. C. Igglesden, Mr. J. Kingsford, Mr. C. H. Read, and Captain Somerset Webb, the retiring governors, have been re-elected governors of the Ashford Cottage Hospital.

The following gentlemen have been elected governors of the Grantham Hospital and members of the managing committee of that institution : The Rev. Canon Welby, the Rev. H. N. Nash, the Rev. H. M. Holden, Messrs. M. G. Thorold, W. E. Dalby, W. Lee, the superintendent Wesleyan minister, and S. L. Williamson.

The following gentlemen have been elected governors of the Scarborough Hospital and Dispensary : Mr. Crowe, Lieut.-Colonel Hill, Mr. Holloway, Archdeacon Lindsay, Mr. A. V. Machin, Mr. J. Taylor, Mr. J. Raynar, and Councillor Thomson, with the addition of Mr. E. Hopper, Mr. T. J. Pearce, the Rev. C. H. Clissold, and Mr. Tetley.

Viscount Tredegar has been re-elected President of the Newport and County Hospital, and the following gentlemen re-elected as governors : Messrs. R. T. Martin, W. A. Baker, C. T. Wallis, the Rev. E. W. Skinner, Messrs. M. Mordey, E. J. Summers, W. Lyndon Moore, H. S. Lyne, A. J. Stevens, J. W. Phillips, F. J. Heybyrne, C. D. Stentiford, the Rev. D. Hickey, Sir George Forestier-Walker, the Rev. A. A. Mathews, Mr. A. A. Newman, the Rev. D. H. Griffiths, Mr. A. H. Laybourne, and the directors on behalf of the Workmen's Hospital Fund.

The Duke of Portland has been unanimously elected President and Mr. W. S. Davy re-elected vice-president of the Newark Hospital.

Westminster Hospital House Committee has elected Sir J. Wolfe Barry Chairman and Mr. W. Vaux Graham Vice-Chairman for the year.

Major Ellis Talbot has been unanimously elected president of the Kidderminster Infirmary and Children's Hospital.

The Governors of the Queen's Hospital for Children, Hackney Road, at their annual meeting, presided over by Lord William Cecil, appointed Lord Shaftesbury to be President of the institution, in succession to the late Lord Amherst of Hackney. Among the Governors present were the Duke of Newcastle (a Vice-President), Dr. Porter Parkinson (senior physician), and Mr. Charles Port (chairman of the House Committee).

The following changes have been made on the administrative staff of the Portsmouth Hospital Saturday Fund : Mr. R. James, owing to advancing age, has resigned the post of honorary treasurer which he has held for twenty-nine years, and has been appointed one of the vice-presidents, Mr. E. Goble succeeding him as hon. treasurer. Mr. Luff was re-elected chairman of committee, and Mr. J. F. Spanner hon. secretary, while the following gentlemen were elected to serve on the Board as governors : Messrs. E. A. Goble, C. Evans, J. Mason, W. Cole, G. Pratt, E. Mead, A. Williams, Preston Watson, and Harris.

During the past year there have been several changes on the committee and administrative staff of the Royal Victoria Hospital at Bournemouth. Mr. H. E. Ludbrook, who was an energetic committee man and took an active interest in the work of the institution, died during the year, and Sir William Karslake, K.C., whose services were equally valuable and appreciated, resigned owing to ill-health. Mr. G. D. Ker, who for years has managed the financial affairs of the hospital as Treasurer, is leaving the town, and has tendered his resignation, which the committee has reluctantly accepted. Another resignation was that of Mr. J. J. Wycliffe-Goodwin, who has acted as Secretary to the hospital, but has lately found it impossible to give all his time to the work. The committee invited applications for the post of Secretary, in consequence of his resignation, and, out of no fewer than 164 candidates, selected Mr. Gordon Martyr. Mr. Martyr was trained at St. Mary's Hospital, London, where for five years he was a member of the secretarial staff, and he took up his new duties on February 1 last.

